# Swedish Child Health Services Register: a quality register for child health services and children’s well-being

**DOI:** 10.1136/bmjpo-2022-001805

**Published:** 2023-01-20

**Authors:** Mattias Wennergren, Karin Berg, Ann-Sofie Frisk Cavefors, Helena Edin, Leif Ekholm, Lars Gelander, Marie Golsäter, Jennie Hedman, Anton Holmgren, Frida Karlsson Videhult, Anna Levin, Sven Arne Silfverdal, Thomas Wallby, Anna Erica Fäldt

**Affiliations:** 1 Research, Education, Development and Innovation, Primary Health Care, Region Västra Götaland, Göteborg, Sweden; 2 Centre of Child Health Services, Regionhälsan, Region Västra Götaland, Göteborg, Sweden; 3 Centre of Child Health Services, Regionhälsan, Region Västra Götaland, Borås, Sweden; 4 Centre of Child Health Services, Department of Children's Health, Academic Hospital, Region Uppsala, Uppsala, Sweden; 5 Centre of Child Health Services, Regional Office, Region Örebro län, Örebro, Sweden; 6 Department of Physiology/Endocrinology, Institute of Neuroscience & Physiology, University of Gothenburg Sahlgrenska Academy, Göteborg, Sweden; 7 Child Health Services, Futurum—Academy for Health and Care, Region Jönköping County, Jönköping, Sweden; 8 CHILD-Research Group, School of Health and Welfare, Jönköping University, Jönköping, Sweden; 9 Local Health Care, Centre of Child Health Services, Region Jämtland Härjedalen, Östersund, Sweden; 10 Department of Pediatrics, Institute of Clinical Sciences, University of Gothenburg Sahlgrenska Academy, Göteborg, Sweden; 11 Department of Pediatrics, Institute of Clinical Sciences, Region Halland, Halmstad, Sweden; 12 Department of Clinical Sciences, Pediatrics, Umeå University, Umeå, Sweden; 13 Competence Centre for Mother and Child Health Care, Regional Office, Region Västerbotten, Umeå, Sweden; 14 Department of Pediatrics, Institute of Clinical Sciences, Region Gotland, Visby, Sweden; 15 Department of Womens and Childrens Health, Uppsala Universitet, Uppsala, Sweden; 16 Department of Public Health and Caring Sciences, Uppsala Universitet, Uppsala, Sweden

**Keywords:** health services research

## Abstract

**Background:**

Swedish child health services (CHS) is a free-of-charge healthcare system that reaches almost all children under the age of 6. The aim for the CHS is to improve children’s physical, psychological and social health by promoting health and development, preventing illness and detecting emerging problems early in the child’s life. The services are defined in a national programme divided into three parts: universal interventions, targeted interventions and indicated interventions.

The Swedish Child Health Services Register (BHVQ) is a national Quality Register developed in 2013. The register extracts data from the child’s health record and automatically presents current data in real time. At present, the register includes 21 variables.

**Aim:**

We aim to describe data available in the BHVQ and the completeness of data in BHVQ across variables.

**Methods:**

Child-specific data were exported from the register, and data for children born in the regions were retrieved from Statistics Sweden to calculate coverage.

**Results:**

The register includes over 110 000 children born between 2011 and 2022 from 221 child healthcare centres in eight of Sweden’s 21 regions. In seven of the eight regions, 100% of centres report data.

The completeness of data differs between participating regions and birth cohorts. The average coverage for children born in 2021 is 71%.

**Conclusions:**

The BHVQ is a valuable resource for evaluating Child Health Services nationally, with high coverage for the youngest children. As a result of continuous improvement of the services, the possibility to follow the development of children’s health in Sweden is possible through the register. When fully expanded, the register will be a natural and essential part of developing preventive services, improving healthcare for children below 6 years of age and a tool for developing evidence-based child health interventions.

**WHAT IS KNOWN ABOUT THE SUBJECT?**

- Swedish child health service aim is to improve children’s physical, psychological and social health by promoting health and development.
- The services are defined in a national programme divided into three parts: universal interventions, targeted interventions and indicated interventions.
- Almost all children in Sweden attend regular visits in the child health services.

**WHAT THIS STUDY ADDS?**

- The Swedish Child Health Services Register has a high coverage among the youngest children in the eight participating regions.
- The register contains data on over 110 000 children; in seven of the eight regions, 100% of centres report data.
- The data in the child health services records are automatically exported to the Swedish Child Health Services Register.

**HOW THIS STUDY MIGHT AFFECT RESEARCH, PRACTICE OR POLICY**

- The Swedish Child Health Services Register is a valuable resource for evaluating methods in used in the Child Health Services.
- Data in the register can be a valuable resource for research regarding children’s health.
- By using personal identification number in the register, it is possible to link data between registries.

## Introduction

Child health services (CHS) in Sweden are free-of-charge and a nationwide healthcare system for children living in Sweden under the age of 6. This covers approximately 710 000 children. The CHS has high credibility in the Swedish community, and almost all children attend at least one regular visit to CHS.^1^ The CHS aims to improve children’s physical, psychological and social health by promoting health and development, preventing illness and detecting emerging problems in the child’s environment. In the CHS, children and families receive preventive healthcare such as vaccinations, parental support and healthy lifestyle promotion. Regular check-ups regarding psychomotor and language development, growth monitoring and vision and hearing screenings are carried out. The services are defined in a national CHS programme divided into three parts: universal interventions, targeted interventions and indicated interventions.^2^ Foremost, the programme is delivered by a registered specialist nurse in primary or paediatric care. The universal part consists of 15–16 health visits, of which 11–12 visits are during the child’s first 12 months. Four team visits with a nurse and a physician, according to the programme, are also included. All the interventions in the programme are based on principles of proportionate universalism. They should be adjusted to the needs of every family^3 4^; thus, the programme can lead to more than 16 visits. The programme is based on the United Nations’ Convention on the Rights of the Child.^5^ The CHS have a unique opportunity to intervene early when staff are concerned about a child’s well-being.^6^

### Child health records and regional child health units

Electronic health records (EHR) have been implemented in the CHS in 20 out of 21 Swedish regions, and the records are well structured, with some regional differences.

In most regions, the staff receive supervision, training, consultation and support in implementing new methods and improvement work from a regional CHS unit, most often consisting of a nurse, a physician and a psychologist. The Swedish CHS has a long tradition of data-driven quality improvement and evaluation processes in which the regional CHS units have a major role. Traditionally, the evaluation of the CHS has been performed through regional annual reports of statistics shared with the CHS providers in dialogue meetings. The statistics have been manually reported by each clinic or extracted from the respective data journal system—often a combination of these methods, thereby these reports have already been outdated when presented due to time lag.

#### The Swedish Child Health Services Register

Based on the need for a national structure for collection of statistical data for quality improvement, the Swedish Child Health Services Register (BHVQ) was the result of an interprofessional national effort to define quality indicators for preventive child healthcare. In 2013, financial support was granted by the Swedish Association of Local Authorities and Regions and a formal board was formed.

BHVQ aims to improve the quality of the care offered within CHS and, as a result, follow the health status of children. Most measurements chosen are healthcare indicators and determinants for children’s long-term health. BHVQ makes it possible to measure health access and outcomes from the CHS programme in both short-term and long-term time horizons.^7^ In the Swedish national system for knowledge-driven management,^8^ BHVQ’s role is to evaluate and deliver data to enable monitoring the national programme nationwide. This facilitates the national system for knowledge-driven management to suggest adding or subtracting indicators in the CHS and BHVQ.

Furthermore, BHVQ is today managed by a register holder and a board of members from different regions in Sweden, including all professions participating in the CHS. Half of the board members have a relevant PhD, and all have a long experience working in the CHS.

#### Regulations and prospects

Sweden has a long history of using and combining data from different registries for scientific purposes.^9^ It is considered a part of the national structure of research and healthcare.^10^ The personal identification number (PIN) is recommended to be used as a base for all registries in Sweden,^11^ which allows data to be combined with other registers and thereafter pseudonymised. The use of PINs and personal data is controlled by regulations regarding the General Data Protection Regulation and Swedish national laws.^12^ Data usage inside national quality registries is regulated in the Swedish patient data act.^13^ For national quality registries, the legislation makes it possible to collect data about patients and patient care with the obligation to inform patients of data collection for a register. The patient can request not to be included in the register and erase data.^13^

### Aim

We aim to describe:

1. Data available in the BHVQ.
2. Completeness of data in BHVQ across variables.

## Methods

For this study, child-specific data were exported from the register’s internal Microsoft Power BI Self-Service portal. Microsoft Power BI calculations were conducted with data extraction from the BHVQ database and analysed and summarised in Microsoft Excel (V.2022). Data for children born in each region were retrieved from Statistics Sweden.

### Variables in the register

BHVQ includes 21 variables, for example, home visits, screening for maternal mental health, growth, language screening and exposure to secondhand smoking, of which most are collected continuously at the visits in the CHS. See online supplemental appendix 1 for a complete list of variables.

10.1136/bmjpo-2022-001805.supp1 Supplementary data

#### Home visits

Two home visits are included in the CHS programme. The first visit is conducted during the first 30 days after birth, and the second around 8 months post-partum.^14^ Home visits can promote childhood development, identify risk factors and improve parenting skills.^15^ BHVQ enables regions to follow the frequency of home visits, allowing for resource allocation. Several projects are ongoing regarding extended home visiting programmes in Sweden.^15–17^ The data structure for the home visits in the BHVQ can be used for collecting data on additional home visits.

#### Parental mental health problems

In BHVQ, participating in the Edinburgh Postnatal Depression Screening^18^ is observed for every child’s mother and reported as a bivariate variable (Have/have not received Edinburgh Postnatal Depression Screening). In most regions, one visit for the non-birth-giving parent is implemented and followed in the BHVQ.

#### Growth

The register monitors all measures on length, height and weight in children at all visits conducted at the CHS.^19^ Body mass index is calculated and presented at three ages: two and a half, four and five.^20^ Since the register follows individual data, further analysis of trajectories is possible.

#### Language screening

The CHS programme includes a language screening at 2.5 or 3 years of age. There are two methods available, and BHVQ provides data usage of both methods.^21 22^

#### Patient-reported measurements

Patient-reported outcome measures are used in BHVQ regarding exposure to secondhand tobacco smoke and the child’s breast milk intake or breast feeding, which both are strongly associated with child health outcomes.^23–26^

### Data collection and storage

The child health records, administrated by the CHS, are the data sources for the register with the child’s PIN as the primary key. Data are automatically exported to the register’s server via a national service-contract platform or point-to-point. As new information is added to the child’s health record, the register is updated. Furthermore, the data are stored on a web platform in a Structured Query Language-Server.

To provide feedback to participating regions, centres and nurses, variables are published online in aggregated form in an interactive report with the possibility of combining data. This enables professionals and decision-makers to follow the quality and production of services in the CHS. A two-step authorisation provides access to detailed data in a specific region.

### Validation of data

To study the validity of the data in the register, a comparison is made with data in the child’s health record in all regions delivering data to the BHVQ, which is audited annually. All variables and their specific values are audited in a specific age category. Age categories 0–12 months, 1–2 years and 3–5 years are used. All variables and values are thereby audited within 3 years.

### Ethical issues and data available for research

All caregivers who visit the CHS with their child receive information that data from the child’s health record are exported to BHVQ, with the possibility to opt out at any time in accordance with current legislation.^13^

After approval from an ethical review board, researchers may obtain pseudonymised data from BHVQ to link to other national and quality registers. This enables following children over time or analysing a single birth cohort.

## Results

### Population

The register aims to include children in Sweden aged 0–5 who have attended CHS and whose caregivers have not opted-out of participation. In January 2023, the register contained data from over 110 000 children born between 2011 and 2022 from participating regions (table 1).

**Table 1:** Number of children in BHVQ and proportion of all children in Sweden according to Statistics Sweden (31 December 2021)

Birth cohort	Total amount of children in the BHVQ N	Percentage of children in Sweden in BHVQ
2011	73	0
2012	530	0
2013	1335	1
2014	3623	3
2015	4984	4
2016	6785	5
2017	14 713	12
2018	15 471	13
2019	14 906	13
2020	16 701	15
2021	17 810	16
2022	14 206	*No denominator available for this birth cohort*
Total	111 137	

BHVQ, The Swedish Child Health Services Register.

### Participating regions

Eight regions in Sweden deliver data from 221 CHS centres. Since 2017, when the region of Dalarna started to deliver data, there have been continuous data transfer projects in several other regions. Hence, the number of children with data in the BHVQ differs between birth cohorts and regions (table 2). In total, 100% of the CHS centres in seven of the eight regions deliver data to the BHVQ.

**Table 2:** Coverage per region in Sweden

Region in Sweden	Start	Birth cohort	Children living in the region N	Children in the BHVQ N (%)
	Year and month for delivering data to BHVQ			
Dalarna	September 2017	2021	2816	2 840 (100)
		2020	2965	3 026 (100)
		2019	3059	2 963 (97)
		2018	3195	2 946 (92)
		2017	3279	2 824 (86)
Gotland	September 2022	2021	532	562 (100)
		2020	496	500 (100)
		2019	566	548 (97)
		2018	585	563 (96)
		2017	637	620 (97)
Gävleborg	December 2021	2021	2770	1 890 (68)
		2020	2815	760 (27)
		2019	2986	439 (15)
		2018	3276	531 (16)
		2017	3099	603 (19)
Jönköping	March 2019	2021	4054	2 514 (62)
		2020	4288	3 757 (88)
		2019	4274	4 020 (94)
		2018	4416	3 843 (87)
		2017	4341	3 147 (72)
Kronoberg	October 2022	2021	2275	1 949 (86)
		2020	2402	1 878 (78)
		2019	2412	1 821 (75)
		2018	2370	1 844 (78)
		2017	2398	1856 (77)
Stockholm	November 2022	2021	28 880	2 905 (10)
		2020	28 288	1 805 (6)
		2019	28 352	2 164 (8)
		2018	28 522	2 470 (9)
		2017	28 968	2 466 (9)
Södermanland	October 2020	2021	3113	3 035 (97)
		2020	3258	3 143 (96)
		2019	3344	2 719 (81)
		2018	3593	2 777 (77)
		2017	3739	2 870 (77)
Västernorrland	October 2022	2021	2393	2 178 (91)
		2020	2325	1 892 (81)
		2019	2531	293 (12)
		2018	2658	545 (21)
		2017	2585	383 (15)

The number of children in each region based on data from Statistics Sweden (31 December 2021) compared with the number of children in the BHVQ per birth cohort. In Stockholm, only one healthcare provider delivers data. In 2023, remaining providers expect to start delivering data.

BHVQ, The Swedish Child Health Services Register.

### Coverage

The completeness of data is compared with data from Statistics Sweden on children born in each region to calculate the coverage (table 2). The completeness of data differs between regions and birth cohorts. The average coverage for children born in 2021 in participating regions is 71%. Since the children must attend the CHS to be included in the register (information obligatory), a relatively higher proportion of the youngest children are included in the register (table 2). Children in the BHVQ include those who have had at least one visit to a specific centre in the region at least once. Families have the right to choose to have their child’s healthcare provided in a different region than the one in which they live. Consequently, a total regional coverage does not indicate that all children living in the region have attended a visit in that specific region.

### Registry data

The registry contains data from visits in the CHS, home visits and health outcomes. Table 3 shows some of the most requested data available in the register. Both data on attended home visits, screenings and health outcomes of these can be exported from the register. Data are reported aggregated, but individual data are available for research. In online supplemental appendix 2, number of children with visits per year and specific age is reported.

10.1136/bmjpo-2022-001805.supp2 Supplementary data

**Table 3:** Results of activities and measurements in the CHS that are exported to the BHVQ

Birth cohort	Received home visits	Received EPDS	Children who got breast milk at 4 months of age	Exposure to secondhand smoke at 8 months of age; mother smokes	Exposure to secondhand smoke at 8 months of age; father smokes	Positive on language screening at 2.5–3 years of age	Negative on language screening at 2.5–3 years of age	No completed language screening at 2.5–3 years of age	Underweight at 2.5–3 years of age	Overweight at 2.5–3 years of age	Obesity at 2.5–3 years of age	Underweight at 4 years of age	Overweight at 4 years of age	Obesity at 4 years of age
2011												3	13	2
2012						20	80	16	1	14	6	3	13	5
2013						17	83	3	1	21	5	1	13	4
2014				3,9	7,1	15	85	6	2	18	4	1	12	4
2015	55	59	72	5,2	8,5	16	84	32	2	17	3	2	10	3
2016	70	65	70	6,2	8,4	14	88	11	2	16	3	2	11	3
2017	75	70	71	3,4	6,5	12	90	26	2	18	4	2	12	4
2018	72	51	72	3,9	7,2	12	88	20	2	17	4	2	10	3
2019	78	64	71	3,6	7,5	13	87	11	2	14	3			
2020	63	79	72	2,9	6,2	16	85	6	1	12	2			
2021	74	76	69	2,7	5,4									
2022	79	73	71	1,6	3,7									

In percentages.

BHVQ, The Swedish Child Health Services Register; CHS, child health services; EPDS, Edinburgh Postnatal Depression Screening.

## Discussion

### BHVQ, as part of the local data-driven quality improvement in the CHS

BHVQ allows the CHS to follow and monitor the CHS programme at every clinic. It also enables an automatic collection of data and a data-driven workflow for the CHS. Through the BHVQ, regions can monitor their data continuously, avoid annual reports with outdated data and compare data nationally. Before BHVQ, annual reports were aggregated regionally with less relevant data for each clinic and could be perceived as criticism and, therefore, tended to be less of a motivation.^27–29^ It could also mean that there was a risk of receiving feedback far too late on negative trends due to outdated data for organisational changes. Using the BHVQ, deviations and variations in the service can be detected early, and relevant interventions can be implemented. The BHVQ provides data to enable data-driven continuous quality improvement within the CHS. With the ability to use the PIN for individuals included in the BHVQ, it is possible to link data to other data sources; moreover, it enables the evaluation of preventive services and their long-term effects. BHVQ can be a tool and a reference for following universal and targeted interventions in the CHS programme.

Several methods used in the CHS programme have limited evidence and are criticised.^30–32^ Using BHVQ methods can be evaluated and gain validity or be discarded from the national CHS programme. BHVQ can therefore be used to develop and evaluate future methods.

### Ethical considerations

Data on healthcare utilisation and health are sensitive and must be secured. In the BHVQ, the PIN is used as a key; however, when data are exported for research, it must be deidentified or pseudo-anonymised. In register research, data shall be handled with discretion. If the data set contains a large amount of data, the possibility to recognise an individual is minimised. All reporting should be aggregated to avoid the possibility of identifying an individual.

### Strengths and limitations

This study is based on data available in the register. Compared nationally, the coverage in the register is low, but from a regional perspective, most younger cohorts have almost complete coverage. Several regions have ongoing projects to deliver data to the BHVQ, and the coverage for every new birth cohort is increasing. Continuous improvements and data validation create opportunities for the register to increase coverage and widen its usage. The difference in coverage between regions that send data to the BHVQ depends on several factors. All guardians must be informed about the BHVQ before data are transferred to the register. During the child’s first year of life, the visits to CHS are more frequent, followed by annual visits. The time delay can be a year or more if the nurse fails to inform the parents about data export to the register. Also, the regional context affects coverage; for example, when EHRs were implemented.

One limitation of this study is that data regarding the quality of CHS and children’s health are not analysed. Further studies are needed to evaluate the CHS and give further recommendations regarding changes in the national CHS programme.

Limitations in the documentation and inconsistent documentation are major challenges for the register, together with several active EHR systems in Sweden. Variations in documentation between regions affect the quality of data. For example, the variable regarding exposure to secondhand smoke differs between regions, where some regions report dichotomous variables, and others only document the presence of exposure. The development of the BHVQ must, therefore, be flexible to include as much data as possible and secure a high-quality level.

## Conclusions

This brief description of the data available in BHVQ shows that the BHVQ is a valuable resource for evaluating CHS at a national level. The completeness of data varies between regions and variables and is increasing as more regions deliver data.

As a result of continuous improvement of the services, the possibility to follow the development of children’s health in Sweden is possible through BHVQ. When fully expanded, the register will be a natural and essential part of developing preventive services, improving healthcare for children below 6 years of age, and a tool for developing evidence-based child health interventions.

Accessing the data online allows decision-makers and healthcare workers to follow and analyse the CHS programme’s outcomes easily. The ambition of the register is that the online platform facilitates resource allocation and improvements to the population’s needs as a data-driven method.

The authors strongly recommend expanding and improving the BHVQ to promote children’s health and develop evidence-based child healthcare.

## Supplementary Material

Author's manuscript

## Data Availability

All data relevant to the study are included in the article or uploaded as supplementary information.
