# Supplementary material for: Swedish Child Health Services Register: a quality register for child health services and children’s well-being
Source: BMJ Paediatr Open. 2023 Jan 20;7(1):e001805. doi: 10.1136/bmjpo-2022-001805 (PMC9872488; doi:10.1136/bmjpo-2022-001805)
Supplement: Supplementary data [file bmjpo-2022-001805supp001.pdf]

Variable	Description	Age of measurements
First born child	Is the child the first born child for any caregiver	0-5 years old
Home visits	Received home visits, per child. Two home visits are a part of the national program from child health care	0-5 years old
Visits at the clinics	Visits at the CHC, per child. Presented per profession the child met at the visits (nurse, physician, paediatrician and GP)	0-5 years old
Team visits at the clinics	Children who meet both a physician and a specialist nurse at the CHC	0-5 years old
Parental support in group	Children whose caregivers participated in a parental support group at the CHC. The groups are usually conducted during the child's first year.	0-5 years old
Postnatal Depression Screening	Children where the mother/birth-giving parent participated in screening for postnatal depression using the Edinburgh Postnatal Depression Scale (EPDS)	6-8 weeks
Individual conversation with the non birth giving caregiver	Children where the father/non-birth-giving parent participated in an individual conversation at the CHC regarding mental health and parenthood	3-5 months
Breastfeeding status	Breastfeeding status during the first year with measurements after 1 week, 2, 4, 6, 8 and 12 months	0-12 months
Vaccinations	Number of doses per child and vaccine in the national vaccination programme for children. This includes vaccinations against rotavirus infection, diphtheria, tetanus, pertussis, polio, Haemophilus influenzae type b, pneumococcal disease, measles, mumps, and rubella. For children "at-risk" the vaccination program also includes selective vaccination against tuberculosis, hepatitis B, influenza and invasive pneumococcal disease, for BHVQ tuberculosis and hepatitis B is included	0-5 years old. National measurement standard at 2 years
Second-hand smoking	Exposure to second hand smoke by caregivers who smokes or the occurrence of smoking in the child’s home	4 weeks, 8 months and 18 months
Growth	Weight, Length/Height, Head Circumference (during the first 18 months), Gestational age at birth and biological parents length. Body mass index (BMI) is calculated at 2,5-3 years, 4 years and 5 years	0-5 years old
Language and communication screening	Completed, no need for assessment and result of screening	2,5-3 years
Audiology screening	Completed, no need for assessment and result of screening	4 years
Ophthalmic screening	Completed, no need for assessment and result of screening	4 years
Developmental assessment	Completed, no need for assessment and result of screening	4 years

Referrals	Referrals send from CHC to audiologist, paediatrician, physiotherapist, speech and language pathologist, orthoptist and psychologist per child	0-5 years old
Date of discharge from delivery ward and maternity ward	Date of discharge from delivery ward and maternity ward	0-3 months
Accidents	Indicators of pre-school children's health	12 months and 4 years
Antibiotics	Indicators of pre-school children's health	12 months and 4 years
Asthma	Indicators of pre-school children's health	12 months and 4 years
Eczema	Indicators of pre-school children's health	12 months and 4 years
