# Supplementary material for: Swedish Child Health Services Register: a quality register for child health services and children’s well-being
Source: BMJ Paediatr Open. 2023 Jan 20;7(1):e001805. doi: 10.1136/bmjpo-2022-001805 (PMC9872488; doi:10.1136/bmjpo-2022-001805)
Supplement: Supplementary data [file bmjpo-2022-001805supp002.pdf]

Appendix 2. The total amount of children in the register with visits at a specific age

Birth Cohort	Total amount of children in the BHVQ	Children with at least one visit at age 0-3 weeks (0–18 days)	Children with at least one visit at age 0-6 weeks (0–42 days)	Children with at least one visit at age 0 weeks - 2 months and 3 weeks (0–82 days)	Children with at least one visit at 4 months of age 105–143 days	Children with at least one visit at 6 months of age (167–213 days)	Children with at least one visit at 8 months of age (227–274 days)	Children with at least one visit at 1,5 years of age (517–638 days)	Children with at least one visit at 2,5-3 years of age (853–1186 days)	Children with at least one visit at 4 years of age (1401–1551 days)	Children with at least one visit at 5 years of age (1766–2100 days)
2011	73									62	73
2012	530		1	1	1	1	1	1	161	409	545
2013	1 335	1	1	1	1	2	1	167	982	1 096	1 304
2014	3 623	78	99	105	116	259	411	1 132	1 382	2 288	3 575
2015	4 984	827	965	1 089	1 171	1 151	1 240	1 395	2 871	4 438	4 786
2016	6 785	1 945	2 094	2 141	1 909	2 058	2 062	2 446	5 345	5 618	6 116
2017	14 713	4 619	5 093	5 274	4 833	5 277	5 554	8 103	10 886	11 585	11 819
2018	15 471	6 541	7 199	7 568	7 503	8 467	8 738	10 373	13 124	11 797	26
2019	14 906	9 543	10 104	10 428	9 737	10 225	10 297	11 715	12 449	28	
2020	16 701	12 973	13 659	13 978	12 942	13 363	13 151	13 685	4 910		
2021	17 810	14 013	15 023	15 641	13 896	14 567	14 493	6 671			
2022	14 206	12 982	13 907	14 145	8 464	6 736	4 154				
Total	111 137	63 522	68 145	70 371	60 573	62 106	60 102	55 688	52 110	37 321	28 244
