# Supplementary material for: Swedish Child Health Services Register: a quality register for child health services and children’s well-being
Source: BMJ Paediatr Open. 2023 Jan 20;7(1):e001805. doi: 10.1136/bmjpo-2022-001805 (PMC9872488; doi:10.1136/bmjpo-2022-001805)
Supplement: Author's manuscript [file bmjpo-2022-001805.draft_revisions.pdf]

BMJ Paediatrics Open

BMJ Paediatrics Open is committed to open peer review. As part of this commitment we make the peer review history of every article we publish publicly available.

When an article is published we post the peer reviewers' comments and the authors' responses online. We also post the versions of the paper that were used during peer review. These are the versions that the peer review comments apply to.

The versions of the paper that follow are the versions that were submitted during the peer review process. They are not the versions of record or the final published versions. They should not be cited or distributed as the published version of this manuscript.

BMJ Paediatrics Open is an open access journal and the full, final, typeset and author-corrected version of record of the manuscript is available on our site with no access controls, subscription charges or pay-per-view fees (http://bmjpaedsopen.bmj.com).

If you have any questions on BMJ Paediatrics Open's open peer review process please email info.bmjpo@bmj.com

BMJ Paediatrics Open

The Swedish Child Health Services Register – a quality register for children's well-being and health equity

Journal:	*BMJ Paediatrics Open*
Manuscript ID	bmjpo-2022-001805
Article Type:	Original research
Date Submitted by the Author:	02-Dec-2022
Complete List of Authors:	Wennergren, Mattias; Region Västra Götaland, Research, Education, Development and Innovation, Primary Health Care; Region Västra Götaland, Regionhälsan, Centre of Child Health Services<br>Berg, Karin; Region Västra Götaland, Regionhälsan, Centre of Child Health Services<br>Cavefors, Ann-Sofie; Region Västra Götaland, Regionhälsan, Centre of Child Health Services<br>Edin, Helena; Region Uppsala, Academic Hospital, Centre of Child Health Services<br>Ekholm, Leif; Region Örebro län, Centre of Child Health Services<br>Gelander, Lars; Region Västra Götaland, Regionhälsan, Centre of Child Health Services; University of Gothenburg Sahlgrenska Academy, Department of Physiology/Endocrinology, Institute of Neuroscience & Physiology<br>Golsäter, Marie; Region Jönköping County, Child Health Services, Futurum - Academy for Health and Care; Jönköping University, CHILD-research group, School of Health and Welfare,<br>Hedman, Jennie; Region Jämtland Härjedalen, Centre of Child Health Services<br>Holmgren, Anton; University of Gothenburg Sahlgrenska Academy, Department of Pediatrics, Institute of Clinical Sciences; Region Halland, Department of Pediatrics, Institute of Clinical Sciences,<br>Karlsson Videhult, Frida; Umeå University, Department of Clinical Sciences, Pediatrics; Region Västerbotten, Competence centre for Mother and Child Health Care, Health Care<br>Levin, Anna; Region Gotland, Department of Pediatrics, Institute of Clinical Sciences,<br>Silverdah, Sven Arne; Umeå University, Department of Clinical Sciences, Pediatrics; Region Västerbotten, Competence centre for Mother and Child Health Care, Health Care<br>Wallby, Thomas; Uppsala Universitet, Department of Womens and Childrens Health<br>Fäldt, Anna; Uppsala Universitet, Dept of Public Health and Caring Sciences
Keywords:	Health services research

I, the Submitting Author has the right to grant and does grant on behalf of all authors of the Work (as defined in the below author licence), an exclusive licence and/or a non-exclusive licence for contributions from authors who are: i) UK Crown employees; ii) where BMJ has agreed a CC-BY licence shall apply, and/or iii) in accordance with the terms applicable for US Federal Government officers or employees acting as part of their official duties; on a worldwide, perpetual, irrevocable, royalty-free basis to BMJ Publishing Group Ltd ("BMJ") its licensees and where the relevant Journal is co-owned by BMJ to the co-owners of the Journal, to publish the Work in this journal and any other BMJ products and to exploit all rights, as set out in our licence.

The Submitting Author accepts and understands that any supply made under these terms is made by BMJ to the Submitting Author unless you are acting as an employee on behalf of your employer or a postgraduate student of an affiliated institution which is paying any applicable article publishing charge ("APC") for Open Access articles. Where the Submitting Author wishes to make the Work available on an Open Access basis (and intends to pay the relevant APC), the terms of reuse of such Open Access shall be governed by a Creative Commons licence – details of these licences and which Creative Commons licence will apply to this Work are set out in our licence referred to above.

Other than as permitted in any relevant BMJ Author's Self Archiving Policies, I confirm this Work has not been accepted for publication elsewhere, is not being considered for publication elsewhere and does not duplicate material already published. I confirm all authors consent to publication of this Work and authorise the granting of this licence.

Confidential: For Review Only

The Swedish Child Health Services Register – a quality register for children's well-being and health equity

Word count: 2432

Confidential: For Review Only

Background: Swedish child health services is a free-of-charge healthcare system that reaches almost all children under the age of six. The aim is to improve children's physical, psychological, and social health by promoting health and development, preventing illness, and detecting emerging problems early in the child's life. The services are defined in a national programme divided into three parts: universal-, targeted- and indicated interventions.

The Swedish Child Health Services Register is a national Quality Register developed in 2013. The register extracts data from the child's health record and automatically presents current data in real-time. At present, the register includes 21 variables.

Methods: Child-specific data was exported from the register, and data for children born in the regions were retrieved from Statistics Sweden.

Results: The register includes 101 953 children born between 2011 to 2022 from 221 child healthcare centres in eight of Sweden's 21 regions. In seven of the eight regions, 100% of centres report data.

The completeness of data differs between regions and birth cohorts. The average coverage for children born in 2021 is 71% (SD 32).

Conclusions: The Swedish Child Health Services Register is a valuable resource for evaluating Child Health Services nationally, with high coverage for the youngest children. As a result of continuous improvement of the services, the possibility to follow the development of children's health in Sweden is possible through the register. When fully expanded, the register will be a natural and essential part of developing preventive services, improving health care for children below six years of age, and a tool for developing evidence-based child health interventions.

Confidential: For Review Only

What is known about the subject?

- Swedish child health service aim is to improve children's physical, psychological, and social health by promoting health and development.
- The services are defined in a national programme divided into three parts: universal-, targeted- and indicated interventions.
- Almost all children in Sweden attend regular visits in the child health services

What this study adds?

- The Swedish Child Health Services Register have a high coverage among the youngest children in the eight participating regions.
- The register contains data on over 100 000 children; in seven of the eight regions, 100% of centres report data.
- The Swedish Child Health Services Register is a valuable resource for evaluating Child Health Services.

Confidential: For Review Only

Introduction

Child health services (CHS) in Sweden are free-of-charge and a nationwide healthcare system for children living in Sweden under the age of six. This covers approximately 710,000 children. The CHS has high credibility in the Swedish community, and almost all children attend at least one regular visit to CHS.[1] The CHS aims to improve children's physical, psychological, and social health by promoting health and development, preventing illness, and detecting emerging problems in the child's environment. In the CHS, children and families receive preventive healthcare such as vaccinations, parental support, and healthy lifestyle promotion. Regular check-ups regarding psychomotor and language development, growth monitoring, and vision and hearing screenings are carried out. The services are defined in a national CHS programme divided into three parts: universal-, targeted- and indicated interventions.[2] Foremost, the programme is delivered by a registered specialist nurse in primary or paediatric care. The universal part consists of 15–16 health visits, of which 11–12 visits are during the child's first 12 months. Four team visits with a nurse and a physician, according to the programme, are also included. All the interventions in the programme are based on principles of proportionate universalism. They should be adjusted to the needs of every family;[3, 4] thus, the programme can lead to more than 16 visits. The programme is based upon the United Nations' Convention on the Rights of the Child[5] as a foundation. The CHS have a unique opportunity to intervene early when staff are concerned about a child's well-being.[6]

Child health records and regional Child health units

Electronic health records (EHR) have been implemented in the CHS in 20 out of 21 Swedish regions, and the records are well structured, with some regional differences.

In most regions, the staff receive supervision, training, consultation, and support in implementing new methods and improvement work from a regional CHS unit, most often consisting of a nurse, a physician, and a psychologist. The Swedish CHS has a long tradition of data-driven quality improvement and evaluation processes in which the regional CHS units have a major role. Traditionally, the evaluation of the CHS has been performed through regional annual reports of statistics shared with the CHS providers in dialogue meetings. The statistics have been manually reported by each clinic or extracted from the respective data journal system – often a combination of these methods. Thereby these reports have already been outdated when presented due to time lag.

The Swedish Child Health Services Register (BHVQ)

Based on the need for a national structure for collection of statistical data for quality development, the Swedish Child Health Services Register (BHVQ) was the result of a multi-year national effort among interprofessional to identify and define quality indicators for preventive child health care. An application for financial support for the development of a National Quality Register was granted in 2013 by the Swedish Association of Local Authorities and Regions and a formal board was formed to work further with the indicators and the technical solutions for data collection.

BHVQ aims to improve the quality of the care offered within CHS and, as a result, follow the health status of children. Most measurements chosen are healthcare indicators and determinants for children's long-term health. BHVQ makes it possible to measure health access and outcomes from the CHS programme in both short-term and long-term time horizons.[4]

Confidential: For Review Only

Furthermore, BHVQ is today managed by a register holder and a board of members from different regions in Sweden, including all professions participating in the CHS. Half of the board members have a relevant PhD, and all have a long experience working in the CHS.

Regulations and prospects

Sweden has a long history of using and combining data from different registries for scientific purposes.[7] It is considered a part of the national structure of research and healthcare.[8] The Personal Identification Number (PIN) is recommended to be used as a base for all registries in Sweden,[9] which allows data to be combined with other registers and thereafter pseudonymised. The use of PINs and a large amount of personal data is also controlled by regulations regarding the General Data Protection Regulation and Swedish national laws, which ensure security and opportunity for every citizen to manage the data collected.[10] Data usage inside national quality registries is also regulated in the Swedish patient data act.[11] For national quality registries, the legislation makes it possible to collect data about patients and patient care with the obligation to inform patients of data collection for a register. The patient can request not to be included in the register and that all data for the patient in the register be erased.[11]

Aim

With this paper, we aim to describe:

1. Data available in the Swedish Child Health Services Register (BHVQ) and
2. Completeness of data in BHVQ across variables

Methods

For this study child-specific data were exported from the register's internal Microsoft Power BI Self-Service portal. Microsoft Power BI calculations were conducted with data extraction from the BHVQ database and analysed and summarised in Microsoft Excel (ver 2022). Data for children born in each region were retrieved from Statistics Sweden.

The study was conducted with ethical approval from the Swedish Ethical Review Authority (Dnr 2021-02905).

Variables in the register

BHVQ includes 21 variables, e.g. home visits, screening for maternal mental health, growth, language screening and exposure to second-hand smoking, of which most are collected continuously at the visits in the CHS. See appendix 1. for a complete list of variables.

Home visits

Two home visits are included in the CHS programme. The first visit is conducted during the first 30 days after birth, and the second around eight months post-partum.[12] Home visits, can promote childhood development, identify risk factors, and improve parenting skills.[13] BHVQ enables regions to follow the frequency of home visits, allowing for resource allocation. Several projects are ongoing regarding extended home visiting programmes in Sweden.[13-15] The data structure for the home visits in the BHVQ can be used for collecting data on additional home visits.

Screening for maternal mental health problems

In BHVQ, participating in the Edinburgh Postnatal Depression Screening[16] is observed for every child's mother and reported as a bivariate variable (Have/have not received EPDS). The outcome from the

Confidential: For Review Only

screening is not presented in BHVQ. In most regions, one visit for the non-birth-giving parent is implemented and followed in the BHVQ.

Growth

The register monitors all measures on length, height and weight in children at all visits conducted at the CHS.[17] Body Mass Index (BMI) is calculated and presented at three ages: two and a half, four, and five.[18] Since the register follows individual data, further analysis of trajectories is possible.

Language screening

The CHS programme includes a language screening at two and a half or three years of age. There are two methods available, and BHVQ provides an data usage of both methods.[19, 20] Additionally, information about referrals to speech and language therapy are collected.

Patient-reported measurements

Patient-reported outcome measures are used in BHVQ regarding exposure to second-hand tobacco smoke and the child's breast milk intake or breastfeeding. Both breast milk and second-hand smoke are strongly associated with child health outcomes.[21-24]

Data collection and storage

The child health records, administrated by the CHS, are the data sources for the register with the child's PIN as the primary key. Data are automatically exported to the register's server via a national service-contract platform or point-to-point. As new information is added to the child's health record, the register is updated. Furthermore, the data are stored on a web platform in a Structured Query Language-Server (SQL-Server) supplied by Carmona, a part of CSAM Carmona AB in Halmstad, Sweden.

To provide feedback to participating regions, centres, and nurses, variables are published online in aggregated form in an interactive report with the possibility of combining registered data. For example, it is possible to follow the growth status and potential referrals for firstborns compared to those who are not first-born. This enables professionals and decision-makers to follow the quality and production of services in the CHS. A two-step authorisation provides access to the data at the right level in the hierarchy for individuals needing to view more detailed data in a specific region.

Validation of data

To study the validity of the data in the register, a comparison is made with data in the child's health record in all regions delivering data to the BHVQ, which is audited annually. All variables and their specific values are audited in a specific age category. Age categories 0–12 months, 1–2 years, and 3–5 years are used. All variables and values are thereby audited within three years.

Ethical issues and data available for research

All caregivers who visit the CHS with their child receive information that data from the child's health record are exported to BHVQ, with the possibility to opt out at any time in accordance with current legislation.[11]

After approval from an ethical review board, researchers may obtain pseudonymised data from BHVQ to link to other national and quality registers. This enables following children over time or analysing a single birth cohort.

Confidential: For Review Only

Results

Population

The register includes all children aged 0–5 who have attended CHS and whose caregivers have not opted-out of participation. In October 2022, the register contained data from almost 100,000 children born between 2011 to 2022.

Participating regions

Eight regions in Sweden deliver data from 221 CHS centres. Since 2017, when the region of Dalarna started to deliver data, there have been continuous data transfer projects in several other regions. Hence, the number of children with data in the BHVQ differs between birth cohorts and regions (Figure 1). In total, 100% of the CHS centres in seven of the eight regions deliver data to the BHVQ.

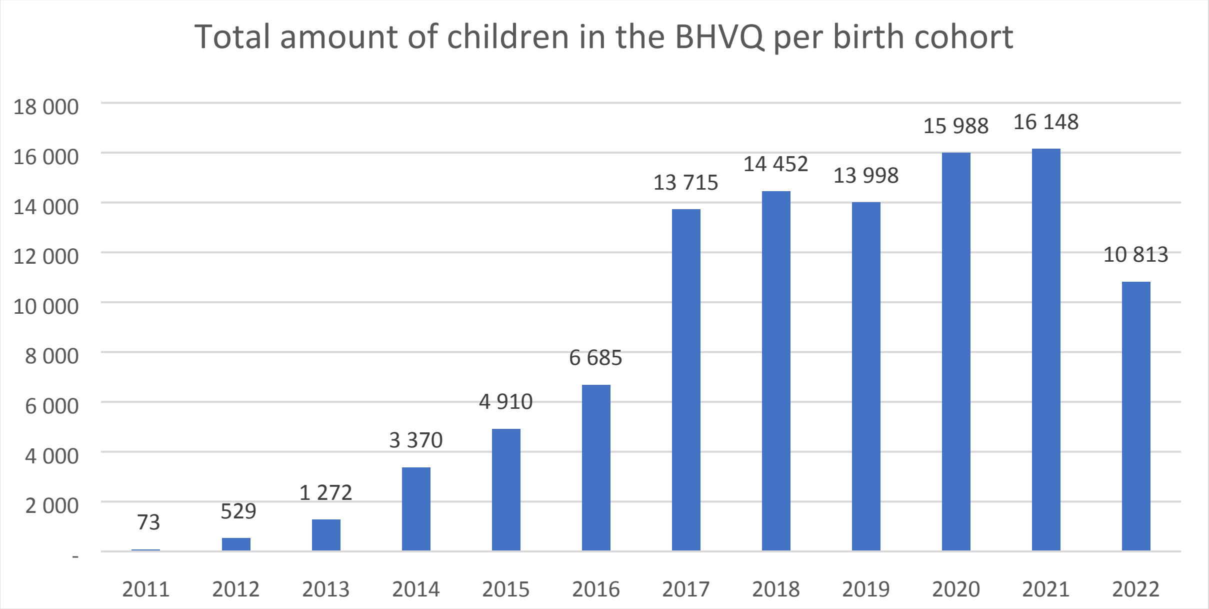

Figure 1. Total number of children in the register by birth cohort

Coverage

The completeness of data is compared with data from Statistics Sweden on children born in each region to calculate the coverage (Table 1). The completeness of data differs between regions and birth cohorts. The average coverage for children born in 2021 is 71% (SD 37). Since the children must attend the CHS to be included in the register (information obligatory), a relatively higher proportion of the youngest children are included in the register (Table 1). Children in the BHVQ include those who have had at least one visit to a specific centre in the region at least once. Families have the right to choose to have their child's health care provided in a different region than the one in which they live. Consequently, a total regional coverage does not indicate that all children living in the region have attended a visit in that specific region.

****** Table 1 about here*****

Table 1. Coverage per Region in Sweden. The number of children in the region compared with the number of children in the BHVQ per birth cohort.

Region in Sweden	Start Year and month	Birth cohort	Children living in the region N	Children in the BHVQ N (%)
**Dalarna**	17-sep	2021	2 816	2836 (100)
		2020	2 965	3016 (100)
		2019	3 059	2954 (97)
		2018	3 195	2937 (92)
		2017	3 279	2817 (86)
**Gotland**	22-sep	2021	532	557 (100)
		2020	496	492 (99)
		2019	566	537 (95)
		2018	585	546 (93)
		2017	637	614 (96)
**Gävleborg**	21-dec	2021	2 770	1863 (67)
		2020	2 815	711 (25)
		2019	2 986	418 (14)
		2018	3 276	471 (14)
		2017	3 099	549 (18)
**Jönköping**	19-mars	2021	4 054	1257 (31)
		2020	4 288	3674 (86)
		2019	4 274	3958 (93)
		2018	4 416	3777 (86)
		2017	4 341	3041 (70)
**Kronoberg**	22-okt	2021	2 275	1744 (77)
		2020	2 402	1548 (64)
		2019	2 412	1643 (68)
		2018	2 370	1473 (62)
		2017	2 398	1543 (64)
**Stockholm**	22-nov	2021	28 880	2724 (9)
		2020	28 288	1539 (5)
		2019	28 352	1484 (5)
		2018	28 522	1989 (7)
		2017	28 968	1930 (7)
**Södermanland**	20-okt	2021	3 113	3020 (97)
		2020	3 258	3126 (96)
		2019	3 344	2693 (81)
		2018	3 593	2741 (76)
		2017	3 739	2834 (76)
**Västernorrland**	22-okt	2021	2 393	2131 (89)
		2020	2 325	1868 (80)
		2019	2 531	231 (9)
		2018	2 658	421 (16)
		2017	2 585	336 (13)

Confidential: For Review Only

Registry data

The registry shows data from child home visits and health outcomes. Table 2 shows some of the most requested data available in the register. Both data on attended home visits, screenings and health outcomes of these can be exported from the register. Data are reported aggregated, but individual data is available for research. In appendix 2 number of children with visits per year and specific age is reported.

Discussion

BHVQ, as part of the local data-driven quality improvement in the CHS

BHVQ allows the CHS to follow and monitor the CHS programme at every clinic. It also enables an automatic collection of data and a data-driven workflow for the CHS. Through the BHVQ, regions can monitor their data continuously, avoid annual reports with outdated data, and compare data nationally. Before BHVQ, annual reports were aggregated regionally with less relevant data for each clinic and could be perceived as criticism and therefore tended to be less of a motivation.[25-27] It also means that there is a risk of receiving feedback far too late on negative trends due to unconscious organisational changes. Using the BHVQ, deviations in the service can be detected early, and relevant interventions can be implemented. The BHVQ provides data to enable data-driven continuous quality improvement within the CHS. With the ability to use the PIN for individuals included in the BHVQ, it is possible to link data to other data sources; moreover, it enables the evaluation of preventive services and their long-term effects. BHVQ can be a tool and a reference for following universal and targeted interventions in the CHS programme.

Ethical considerations

Data on health care utilisation and health are sensitive and must be secured. In the BHVQ, the PIN is used as a key; however, when data are exported for research, it must be de-identified or pseudo-anonymised. In register research, data shall be handled with discretion. If the data set contains a large amount of data, the possibility to recognise an individual is minimised. All reporting should be aggregated to avoid the possibility of identifying an individual.

Strengths and Limitations

This study is based on data available in the register. Compared nationally, the coverage in the register is low, but from a regional perspective, most younger cohorts have almost complete coverage. Several regions have ongoing projects to deliver data to the BHVQ, and the coverage for every new birth cohort is increasing. Continuous improvements and data validation create opportunities for the register to increase coverage and widen its usage. The difference in coverage between regions that send data to the BHVQ depends on several factors. All guardians must be informed about the BHVQ before data are transferred to the register. During the child's first year of life, the visits to CHS are more frequent, followed by annual visits. The time delay can be a year or more if the nurse fails to inform the parents about data export to the register. Also, the regional context affects coverage; for example, when electronic health records were implemented.

Limitations in the documentation and inconsistent documentation are major challenges for the register, together with several active EHR systems in Sweden. Variations in documentation between regions affect the quality of data. For example, the variable regarding exposure to second-hand smoke differs between regions, where some regions report dichotomous variables, and others only

Confidential: For Review Only

document the presence of exposure. The development of the BHVQ must therefore be flexible to include as much data as possible and secure a high-quality level.

Conclusions

This brief description presents a valuable resource for evaluating CHS at a national level. As a result of continuous improvement of the services, the possibility to follow the development of children's health in Sweden is possible through BHVQ. When fully expanded, the register will be a natural and essential part of developing preventive services, improving health care for children below six years of age, and a tool for developing evidence-based child health interventions.

Accessing the data online allows decision-makers and healthcare workers to follow and analyse the CHS programme's outcomes easily. The ambition of the register is that the online platform facilitates resource allocation and improvements to the population's needs as a data-driven method.

The authors strongly recommend expanding and improving the BHVQ to promote children's health and develop evidence-based child health care.

Author contribution

MW and AF conceived the study and its design and performed the analyses. MW and AF prepared the draft and AC, AH, AL, FKV, HE, JH, KB, LE, LG MG, SA and TW provided critical revisions of the intellectual content. All authors read and approved the final manuscript.

Funding

The authors have not declared a specific grant for this research from any funding agency in the public, commercial or not-for-profit sectors.

Declaration of interest statement

The authors have no conflicts of interest to declare.

Patient and public involvement

Patients and/or the public were not involved in the design, or conduct, or reporting, or dissemination plans of this research.

The authors would like to thank Meena Strömqvist at Strömqvist Legal & Language Services AB for a good and quick language check.

Confidential: For Review Only

References

1. Wallby T and Hjern A. Child health care uptake among low-income and immigrant families in a Swedish county. *Acta Paediatrica* 2011; 100: 1495-1503. DOI: 10.1111/j.1651-2227.2011.02344.x.
2. Reuter A. Barnhälsovårdens nationella program, https://www.rikshandboken-bhv.se/metoder--riktlinjer/barnhalsovardens-nationella-program/ (2018, accessed 2022-08-09).
3. Nygren US, Sandberg H, Tindberg Y, et al. Perceived needs for team-based visits in Swedish child healthcare services exceed its existence-A mixed-methods study targeting healthcare professionals. *Acta Paediatr* 2022; 111: 653-666. 2021/11/24. DOI: 10.1111/apa.16193.
4. Socialstyrelsen. Vägledning för barnhälsovården. 2014, p. 172.
5. Assembly UNG. Convention on the Rights of the Child. In: Nations U, (ed.). United Nations, Treaty Series1989.
6. Västra Götalandsregionen CB. Årsrapport Barnhälsovård i Västra Götaland. In: Barnhälsovård C, (ed.). Göteborg2018, p. 52.
7. Rosén ME, A. Dataregister i vården en kollektiv nyttighet. *Läkartidningen* 1999; 96: 6.
8. Laugesen K, Ludvigsson JF, Schmidt M, et al. Nordic Health Registry-Based Research: A Review of Health Care Systems and Key Registries. *Clin Epidemiol* 2021; 13: 533-554. 2021/07/30. DOI: 10.2147/CLEP.S314959.
9. Ludvigsson JF, Otterblad-Olausson P, Pettersson BU, et al. The Swedish personal identity number: possibilities and pitfalls in healthcare and medical research. *Eur J Epidemiol* 2009; 24: 659-667. 2009/06/09. DOI: 10.1007/s10654-009-9350-y.
10. Regulation (EU) 2016/679 of the European Parliament and of the Council of 27 April 2016 on the protection of natural persons with regard to the processing of personal data and on the free movement of such data, and repealing Directive 95/46/EC (General Data Protection Regulation). *Official journal of the European Union*; 59.
11. Patientdatalag (2008:355). In: Socialdepartementet, (ed.). Svensk Författningssamling (SFS).
12. Lindfors A and Almqvist-Tangen G. Hembesök - Rikshandboken för Barnhälsovård, https://www.rikshandboken-bhv.se/metoder--riktlinjer/hembesok/ (2021, accessed 2022-06-01).
13. Barboza M, Kulane A, Burstrom B, et al. A better start for health equity? Qualitative content analysis of implementation of extended postnatal home visiting in a disadvantaged area in Sweden. *Int J Equity Health* 2018; 17: 42. 2018/04/11. DOI: 10.1186/s12939-018-0756-6.
14. Barboza M, Marttila A, Burstrom B, et al. Towards health equity: core components of an extended home visiting intervention in disadvantaged areas of Sweden. *BMC Public Health* 2022; 22: 1091. 2022/06/02. DOI: 10.1186/s12889-022-13492-3.
15. Burström B, Marttila A, Kulane A, et al. Practising proportionate universalism - a study protocol of an extended postnatal home visiting programme in a disadvantaged area in Stockholm, Sweden. *BMC Health Serv Res* 2017; 17: 91. 2017/01/29. DOI: 10.1186/s12913-017-2038-1.
16. Cox JL, Holden JM and Sagovsky R. Detection of postnatal depression. Development of the 10-item Edinburgh Postnatal Depression Scale. *Br J Psychiatry* 1987; 150: 782-786. 1987/06/01. DOI: 10.1192/bjp.150.6.782.
17. Derwig M. Instruktioner för mätning och vägning på BVC, https://www.rikshandboken-bhv.se/tillvaxt--utveckling/tillvaxt/instruktioner-for-matning-och-vagning-pa-bvc/ (accessed 2022-09-05).
18. Cole TJ and Lobstein T. Extended international (IOTF) body mass index cut-offs for thinness, overweight and obesity. *Pediatr Obes* 2012; 7: 284-294. DOI: 10.1111/j.2047-6310.2012.00064.x.
19. Mattsson CM, Marild S and Pehrsson NG. Evaluation of a language-screening programme for 2.5-year-olds at Child Health Centres in Sweden. *Acta Paediatrica* 2001; 90: 339-344.

Confidential: For Review Only

20. Westerlund M and Sundelin C. Can severe language disability be identified in three-year-olds? Evaluation of a routine screening procedure. *Acta Paediatrica* 2000; 89: 94-100. 2000/02/17.
21. Cook DG and Strachan DP. Health effects of passive smoking-10: Summary of effects of parental smoking on the respiratory health of children and implications for research. *Thorax* 1999; 54: 357-366. 1999/03/27. DOI: 10.1136/thx.54.4.357.
22. Makadia LD, Roper PJ, Andrews JO, et al. Tobacco Use and Smoke Exposure in Children: New Trends, Harm, and Strategies to Improve Health Outcomes. *Curr Allergy Asthma Rep* 2017; 17: 55. 2017/07/26. DOI: 10.1007/s11882-017-0723-0.
23. Wallby T and Hjern A. Parental region of birth, socio-economic status and infants' exposure to second-hand smoke. *Acta Paediatr* 2008; 97: 1542-1545. 2008/08/16. DOI: 10.1111/j.1651-2227.2008.00964.x.
24. Meek JY, Noble L and Section on B. Policy Statement: Breastfeeding and the Use of Human Milk. *Pediatrics* 2022; 150 2022/08/04. DOI: 10.1542/peds.2022-057988.
25. Arvidsson E, Dahlin S and Anell A. Conditions and barriers for quality improvement work: a qualitative study of how professionals and health centre managers experience audit and feedback practices in Swedish primary care. *BMC Fam Pract* 2021; 22: 113. 2021/06/16. DOI: 10.1186/s12875-021-01462-4.
26. Regeringskansliet. SOU 2021:34, Börja med barnen! - En sammanhållen god och nära vård för barn och unga. Delbetänkande av Utredningen om en sammanhållen god och nära vård för barn och unga. In: Socialdepartementet, (ed.). Stockholm: Statens offentliga utredningar, 2021, p. 684.
27. Regeringskansliet. SOU 2021:78, Börja med barnen! - Följa upp hälsa och dela information för en god och nära vård. Slutbetänkande av Utredningen om en sammanhållen god och nära vård för barn och unga. In: Socialdepartementet, (ed.). Stockholm: Statens offentliga utredningar, 2021, p. 272.

Confidential: For Review Only

Table 2. Results of activities and measurements in the CHS that are exported to the BHVQ. In percentages

Birth Cohort	Received home visits	Received EPDS	Children who got breast milk at 4 months of age	Exposure to second hand smoke at 8 months of age; mother smokes	Exposure to second hand smoke at 8 months of age; father smokes	Abnormal language screening results at 2.5–3 years of age	Normal language screening results at 2.5–3 years of age	Under weight at 2.5–3 years of age	Over-weight at 2.5–3 years of age	Obesity at 2.5–3 years of age	Under weight at 4 years of age	Over-weight at 4 years of age	Obesity at 4 years of age
2011											3	13	2
2012						17	67	1	14	6	3	13	5
2013						16	81	1	21	5	1	13	4
2014				4	7	15	79	2	17	4	1	12	4
2015	57	62	72	5	9	16	79	2	17	3	2	10	3
2016	71	66	70	6	8	11	68	2	16	3	1	11	3
2017	74	68	71	4	7	9	69	2	18	4	2	12	4
2018	79	57	72	4	7	10	73	2	17	4	2	10	3
2019	78	63	71	4	8	12	77	2	14	3			
2020	62	78	71	3	6	15	78	1	12	2			
2021	73	74	68	3	5								
2022	77	70	69	1	4								

Confidential: For Review Only

Variable	Description	Age of measurements
First born child	Is the child the first born child for any caregiver	0-5 years old
Home visits	Received home visits, per child. Two home visits are a part of the national program from child health care	0-5 years old
Visits at the clinics	Visits at the CHC, per child. Presented per profession the child met at the visits (nurse, physician, paediatrician and GP)	0-5 years old
Team visits at the clinics	Children who meet both a physician and a specialist nurse at the CHC	4 weeks, 6 months, 12 months and 2,5-3 years
Parental support in group	Children whose caregivers participated in a parental support group at the CHC. The groups are usually conducted during the child's first year.	0-5 years old
Postnatal Depression Screening	Children where the mother/birth-giving parent participated in screening for postnatal depression using the Edinburgh Postnatal Depression Scale (EPDS)	6-8 weeks
Individual conversation with the non birth giving caregiver	Children where the father/non-birth-giving parent participated in an individual conversation at the CHC regarding mental health and parenthood	3-5 months
Breastfeeding status	Breastfeeding status during the first year with measurements after 1 week, 2, 4, 6, 8 and 12 months	0-12 months
Vaccinations	Number of doses per child and vaccine in the national vaccination programme for children. This includes vaccinations against rotavirus infection, diphtheria, tetanus, pertussis, polio, Haemophilus influenzae type b, pneumococcal disease, measles, mumps, and rubella. For children "at-risk" the vaccination program also includes selective vaccination against tuberculosis, hepatitis B, influenza and invasive pneumococcal disease, for BHVQ tuberculosis and hepatitis B is included	0-5 years old. National measurement standard at 2 years
Second-hand smoking	Exposure to second hand smoke by caregivers who smokes or the occurrence of smoking in the child's home	4 weeks, 8 months and 18 months
Growth	Weight, Length/Height, Head Circumference (during the first 18 months), Gestational age at birth and biological parents length. Body mass index (BMI) is calculated at 2,5-3 years, 4 years and 5 years	0-5 years old
Language and communication screening	Completed, no need for assessment and result of screening	2,5-3 years
Audiology screening	Completed, no need for assessment and result of screening	4 years
Ophthalmic screening	Completed, no need for assessment and result of screening	4 years
Developmental assessment	Completed, no need for assessment and result of screening	4 years

Confidential: For Review Only

Referrals	Referrals send from CHC to audiologist, paediatrician, physiotherapist, speech and language pathologist, orthoptist and psychologist per child	0-5 years old
Date of discharge from delivery ward and maternity ward	Date of discharge from delivery ward and maternity ward	0-3 months
Accidents	Indicators of pre-school children's health	12 months and 4 years
Antibiotics	Indicators of pre-school children's health	12 months and 4 years
Asthma	Indicators of pre-school children's health	12 months and 4 years
Eczema	Indicators of pre-school children's health	12 months and 4 years

Confidential: For Review Only

Appendix 2. The total amount of children in the register with visits at a specific age

Birth Cohort	Total amount of children in the BHVQ	Children with at least one visit at age 0–18 days	Children with at least one visit at age 0–42 days	Children with at least one visit at age 0–82 days	Children with at least one visit at age 105–143 days	Children with at least one visit at age 167–213 days	Children with at least one visit at age 227–274 days	Children with at least one visit at age 517–638 days	Children with at least one visit at age 853–1186 days	Children with at least one visit at age 1401–1551 days	Children with at least one visit at age 1766–2100 days
2011	73									62	73
2012	529								160	408	544
2013	1 272							165	979	1 093	1 266
2014	3 370			1	14	156	313	1 021	1 257	1 302	3 400
2015	4 910	802	927	1 051	1 134	1 114	1 203	1 355	1 801	4 033	4 701
2016	6 685	1 916	2 059	2 106	1 880	2 025	2 026	2 367	4 458	5 507	4 822
2017	13 715	4 229	4 670	4 844	4 435	4 837	4 846	6 410	10 255	9 696	8 180
2018	14 452	5 309	5 702	5 920	5 948	6 975	7 640	9 691	12 119	8 303	6
2019	13 998	9 120	9 706	10 074	9 442	9 868	9 980	11 097	9 479		
2020	15 988	12 474	13 136	13 409	12 402	12 630	12 209	11 183	2 885		
2021	16 148	12 501	13 410	13 991	11 976	12 199	12 247	4 246			
2022	10 813	9 718	10 422	10 619	5 771	4 007	2 211				
**Total**	**101 953**	**56 069**	**60 032**	**62 015**	**53 002**	**53 811**	**52 675**	**47 535**	**43 393**	**30 404**	**22 992**

Confidential: For Review Only

BMJ Paediatrics Open

The Swedish Child Health Services Register – a quality register for child health services and children's well-being

Journal:	*BMJ Paediatrics Open*
Manuscript ID	bmjpo-2022-001805.R1
Article Type:	Original research
Date Submitted by the Author:	04-Jan-2023
Complete List of Authors:	Wennergren, Mattias; Region Västra Götaland, Research, Education, Development and Innovation, Primary Health Care; Region Västra Götaland, Regionhälsan, Centre of Child Health Services<br>Berg, Karin; Region Västra Götaland, Regionhälsan, Centre of Child Health Services<br>Cavefors, Ann-Sofie; Region Västra Götaland, Regionhälsan, Centre of Child Health Services<br>Edin, Helena; Region Uppsala, Academic Hospital, Centre of Child Health Services<br>Ekholm, Leif; Region Örebro län, Centre of Child Health Services<br>Gelander, Lars; Region Västra Götaland, Regionhälsan, Centre of Child Health Services; University of Gothenburg Sahlgrenska Academy, Department of Physiology/Endocrinology, Institute of Neuroscience & Physiology<br>Golsäter, Marie; Region Jönköping County, Child Health Services, Futurum - Academy for Health and Care; Jönköping University, CHILD-research group, School of Health and Welfare,<br>Hedman, Jennie; Region Jämtland Härjedalen, Centre of Child Health Services<br>Holmgren, Anton; University of Gothenburg Sahlgrenska Academy, Department of Pediatrics, Institute of Clinical Sciences; Region Halland, Department of Pediatrics, Institute of Clinical Sciences,<br>Karlsson Videhult, Frida; Umeå University, Department of Clinical Sciences, Pediatrics; Region Västerbotten, Competence centre for Mother and Child Health Care, Health Care<br>Levin, Anna; Region Gotland, Department of Pediatrics, Institute of Clinical Sciences,<br>Silverdah, Sven Arne; Umeå University, Department of Clinical Sciences, Pediatrics; Region Västerbotten, Competence centre for Mother and Child Health Care, Health Care<br>Wallby, Thomas; Uppsala Universitet, Department of Womens and Childrens Health<br>Fäldt, Anna; Uppsala Universitet, Dept of Public Health and Caring Sciences
Keywords:	Health services research

SCHOLARONE™
Manuscripts

I, the Submitting Author has the right to grant and does grant on behalf of all authors of the Work (as defined in the below author licence), an exclusive licence and/or a non-exclusive licence for contributions from authors who are: i) UK Crown employees; ii) where BMJ has agreed a CC-BY licence shall apply, and/or iii) in accordance with the terms applicable for US Federal Government officers or employees acting as part of their official duties; on a worldwide, perpetual, irrevocable, royalty-free basis to BMJ Publishing Group Ltd ("BMJ") its licensees and where the relevant Journal is co-owned by BMJ to the co-owners of the Journal, to publish the Work in this journal and any other BMJ products and to exploit all rights, as set out in our licence.

The Submitting Author accepts and understands that any supply made under these terms is made by BMJ to the Submitting Author unless you are acting as an employee on behalf of your employer or a postgraduate student of an affiliated institution which is paying any applicable article publishing charge ("APC") for Open Access articles. Where the Submitting Author wishes to make the Work available on an Open Access basis (and intends to pay the relevant APC), the terms of reuse of such Open Access shall be governed by a Creative Commons licence – details of these licences and which Creative Commons licence will apply to this Work are set out in our licence referred to above.

Other than as permitted in any relevant BMJ Author's Self Archiving Policies, I confirm this Work has not been accepted for publication elsewhere, is not being considered for publication elsewhere and does not duplicate material already published. I confirm all authors consent to publication of this Work and authorise the granting of this licence.

Confidential: For Review Only

The Swedish Child Health Services Register – a quality register for child health services and children's well-being

Mattias Wennergren[1, 2], Karin Berg[3], Ann-Sofie Cavefors[2], Helena Edin[4], Leif Ekholm[5], Lars Gelander[3, 6], PhD, Marie Golsäter[7, 8], PhD, Jennie Hedman[9], Anton Holmgren[10, 11], PhD, Frida Karlsson Videhult[12], PhD, Anna Levin[13], Sven-Arne Silfverdal, PhD, MPH[, 12,14], Thomas Wallby[15], PHD, Anna Fäldt[16], PhD

Corresponding author and request for offprints: Anna Fäldt Uppsala University, Child Health and Parenting (CHAP), Department of Public Health and Caring Sciences, Uppsala University, Uppsala, Sweden, Box 564, 751 22 UPPSALA anna.faldt@pubcare.uu.se, Telephone: +46 76 776 77 55

Affiliations

1. Region Västra Götaland, Research, Education, Development and Innovation, Primary Health Care, Gothenburg, Sweden
2. Region Västra Götaland, Regionhälsan, Centre of Child Health Services, Gothenburg, Sweden
3. Region Västra Götaland, Regionhälsan, Centre of Child Health Services, Borås, Sweden
4. Region Örebro Län, Centre of Child Health Services, Örebro, Sweden
5. Department of Physiology/Endocrinology, Institute of Neuroscience & Physiology, Sahlgrenska Academy, University of Gothenburg, Gothenburg, Sweden
6. Child Health Services, Futurum - Academy for Health and Care, Region Jönköping County, Jönköping, Region Jönköping County, Jönköping, Sweden
7. CHILD -research group, School of Health and Welfare, Jönköping University, Jönköping, Sweden
8. Region Jämtland-Härjedalen, Centre of Child Health Services, Östersund, Sweden
9. Department of Pediatrics, Institute of Clinical Sciences, The Sahlgrenska Academy, University of Gothenburg, Gothenburg, Sweden
10. Department of Pediatrics, Halland Hospital, Halmstad, Sweden
11. Competence centre for Mother and Child Health Care, Region Västerbotten, Umeå Sweden
12. Region Gotland, Department of Pediatrics, Centre of Child Health Services, Visby, Sweden
13. Umeå University, Department of Clinical Sciences, Pediatrics, Umeå Sweden
14. Uppsala University, Department of Womens and Childrens Health, Uppsala, Sweden
15. Uppsala University, Child Health and Parenting (CHAP), Department of Public Health and Caring Sciences, Uppsala University

Word count: 2498

Confidential: For Review Only

Background: Swedish child health services is a free-of-charge healthcare system that reaches almost all children under the age of six. The aim is to improve children's physical, psychological, and social health by promoting health and development, preventing illness, and detecting emerging problems early in the child's life. The services are defined in a national programme divided into three parts: universal-, targeted- and indicated interventions.

The Swedish Child Health Services Register is a national Quality Register developed in 2013. The register extracts data from the child's health record and automatically presents current data in real-time. At present, the register includes 21 variables.

Aim: With this paper, we aim to describe data available in the Swedish Child Health Services Register (BHVQ) and the completeness of data in BHVQ across variables.

Methods: Child-specific data was exported from the register, and data for children born in the regions were retrieved from Statistics Sweden to calculate coverage.

Results: The register includes over 110,000 children born between 2011 to 2022 from 221 child healthcare centres in eight of Sweden's 21 regions. In seven of the eight regions, 100% of centres report data.

The completeness of data differs between regions and birth cohorts. The average coverage for children born in 2021 is 71%.

Conclusions: The Swedish Child Health Services Register is a valuable resource for evaluating Child Health Services nationally, with high coverage for the youngest children. As a result of continuous improvement of the services, the possibility to follow the development of children's health in Sweden is possible through the register. When fully expanded, the register will be a natural and essential part of developing preventive services, improving health care for children below six years of age, and a tool for developing evidence-based child health interventions.

Confidential: For Review Only

What is known about the subject?

- Swedish child health service aim is to improve children's physical, psychological, and social health by promoting health and development.
- The services are defined in a national programme divided into three parts: universal-, targeted- and indicated interventions.
- Almost all children in Sweden attend regular visits in the child health services

What this study adds?

- The Swedish Child Health Services Register have a high coverage among the youngest children in the eight participating regions.
- The register contains data on over 110,000 children; in seven of the eight regions, 100% of centres report data.
- The data in the child health services records are automatically exported to the Swedish Child Health Services Register.

How this study might affect research, practice or policy – summarise the implications of this study

- The Swedish Child Health Services Register is a valuable resource for evaluating methods in used in the Child Health Services.
- Data in the register can be a valuable resource for research regarding children's health
- By using personal identification number in the register, it is possible to link data between registries.

Confidential: For Review Only

Introduction

Child health services (CHS) in Sweden are free-of-charge and a nationwide healthcare system for children living in Sweden under the age of six. This covers approximately 710,000 children. The CHS has high credibility in the Swedish community, and almost all children attend at least one regular visit to CHS.[1] The CHS aims to improve children's physical, psychological, and social health by promoting health and development, preventing illness, and detecting emerging problems in the child's environment. In the CHS, children and families receive preventive healthcare such as vaccinations, parental support, and healthy lifestyle promotion. Regular check-ups regarding psychomotor and language development, growth monitoring, and vision and hearing screenings are carried out. The services are defined in a national CHS programme divided into three parts: universal-, targeted- and indicated interventions.[2] Foremost, the programme is delivered by a registered specialist nurse in primary or paediatric care. The universal part consists of 15–16 health visits, of which 11–12 visits are during the child's first 12 months. Four team visits with a nurse and a physician, according to the programme, are also included. All the interventions in the programme are based on principles of proportionate universalism. They should be adjusted to the needs of every family;[3, 4] thus, the programme can lead to more than 16 visits. The programme is based upon the United Nations' Convention on the Rights of the Child[5] as a foundation. The CHS have a unique opportunity to intervene early when staff are concerned about a child's well-being.[6]

Child health records and regional Child health units

Electronic health records (EHR) have been implemented in the CHS in 20 out of 21 Swedish regions, and the records are well structured, with some regional differences.

In most regions, the staff receive supervision, training, consultation, and support in implementing new methods and improvement work from a regional CHS unit, most often consisting of a nurse, a physician, and a psychologist. The Swedish CHS has a long tradition of data-driven quality improvement and evaluation processes in which the regional CHS units have a major role. Traditionally, the evaluation of the CHS has been performed through regional annual reports of statistics shared with the CHS providers in dialogue meetings. The statistics have been manually reported by each clinic or extracted from the respective data journal system – often a combination of these methods. Thereby these reports have already been outdated when presented due to time lag.

The Swedish Child Health Services Register (BHVQ)

Based on the need for a national structure for collection of statistical data for quality improvement, the Swedish Child Health Services Register (BHVQ) was the result of an interprofessional national effort to define quality indicators for preventive child health care. In 2013 financial support was granted by the Swedish Association of Local Authorities and Regions and a formal board was formed.

BHVQ aims to improve the quality of the care offered within CHS and, as a result, follow the health status of children. Most measurements chosen are healthcare indicators and determinants for children's long-term health. BHVQ makes it possible to measure health access and outcomes from the CHS programme in both short-term and long-term time horizons.[7] In the Swedish national system for knowledge-driven management,[8] BHVQ's role is to evaluate and deliver data to enable monitoring the national program nationwide. This facilitates the national system for knowledge-driven management to suggest adding or subtracting indicators in the CHS and BHVQ.

Confidential: For Review Only

Furthermore, BHVQ is today managed by a register holder and a board of members from different regions in Sweden, including all professions participating in the CHS. Half of the board members have a relevant PhD, and all have a long experience working in the CHS.

Regulations and prospects

Sweden has a long history of using and combining data from different registries for scientific purposes.[9] It is considered a part of the national structure of research and healthcare.[10] The Personal Identification Number (PIN) is recommended to be used as a base for all registries in Sweden,[11] which allows data to be combined with other registers and thereafter pseudonymised. The use of PINs and personal data is also controlled by regulations regarding the General Data Protection Regulation and Swedish national laws.[12] Data usage inside national quality registries is regulated in the Swedish patient data act.[13] For national quality registries, the legislation makes it possible to collect data about patients and patient care with the obligation to inform patients of data collection for a register. The patient can request not to be included in the register and erase data.[13]

Aim

With this paper, we aim to describe:

1. Data available in the Swedish Child Health Services Register (BHVQ) and
2. Completeness of data in BHVQ across variables

Methods

For this study child-specific data were exported from the register's internal Microsoft Power BI Self-Service portal. Microsoft Power BI calculations were conducted with data extraction from the BHVQ database and analysed and summarised in Microsoft Excel (ver 2022). Data for children born in each region were retrieved from Statistics Sweden.

The study was conducted with ethical approval from the Swedish Ethical Review Authority (Dnr 2021-02905).

Variables in the register

BHVQ includes 21 variables, e.g. home visits, screening for maternal mental health, growth, language screening and exposure to second-hand smoking, of which most are collected continuously at the visits in the CHS. See appendix 1. for a complete list of variables.

Home visits

Two home visits are included in the CHS programme. The first visit is conducted during the first 30 days after birth, and the second around eight months post-partum.[14] Home visits, can promote childhood development, identify risk factors, and improve parenting skills.[15] BHVQ enables regions to follow the frequency of home visits, allowing for resource allocation. Several projects are ongoing regarding extended home visiting programmes in Sweden.[15-17] The data structure for the home visits in the BHVQ can be used for collecting data on additional home visits.

Parental mental health problems

In BHVQ, participating in the Edinburgh Postnatal Depression Screening[18] is observed for every child's mother and reported as a bivariate variable (Have/have not received EPDS). In most regions, one visit for the non-birth-giving parent is implemented and followed in the BHVQ.

Confidential: For Review Only

Growth

The register monitors all measures on length, height and weight in children at all visits conducted at the CHS.[19] Body Mass Index (BMI) is calculated and presented at three ages: two and a half, four, and five.[20] Since the register follows individual data, further analysis of trajectories is possible.

Language screening

The CHS programme includes a language screening at two and a half or three years of age. There are two methods available, and BHVQ provides data usage of both methods.[21, 22]

Patient-reported measurements

Patient-reported outcome measures are used in BHVQ regarding exposure to second-hand tobacco smoke and the child's breast milk intake or breastfeeding, which both are strongly associated with child health outcomes.[23-26]

Data collection and storage

The child health records, administrated by the CHS, are the data sources for the register with the child's PIN as the primary key. Data are automatically exported to the register's server via a national service-contract platform or point-to-point. As new information is added to the child's health record, the register is updated. Furthermore, the data are stored on a web platform in a Structured Query Language-Server (SQL-Server).

To provide feedback to participating regions, centres, and nurses, variables are published online in aggregated form in an interactive report with the possibility of combining data. This enables professionals and decision-makers to follow the quality and production of services in the CHS. A two-step authorisation provides access to detailed data in a specific region.

Validation of data

To study the validity of the data in the register, a comparison is made with data in the child's health record in all regions delivering data to the BHVQ, which is audited annually. All variables and their specific values are audited in a specific age category. Age categories 0–12 months, 1–2 years, and 3–5 years are used. All variables and values are thereby audited within three years.

Ethical issues and data available for research

All caregivers who visit the CHS with their child receive information that data from the child's health record are exported to BHVQ, with the possibility to opt out at any time in accordance with current legislation.[13]

After approval from an ethical review board, researchers may obtain pseudonymised data from BHVQ to link to other national and quality registers. This enables following children over time or analysing a single birth cohort.

Results

Population

The register aims to include children in Sweden aged 0–5 who have attended CHS and whose caregivers have not opted-out of participation. In January 2023, the register contained data from over 110,000 children born between 2011 to 2022 from participating regions (Table 1).

Confidential: For Review Only

Participating regions

Eight regions in Sweden deliver data from 221 CHS centres. Since 2017, when the region of Dalarna started to deliver data, there have been continuous data transfer projects in several other regions. Hence, the number of children with data in the BHVQ differs between birth cohorts and regions (Table 2). In total, 100% of the CHS centres in seven of the eight regions deliver data to the BHVQ.

Table 1. Number of children in BHVQ and proportion of all children in Sweden according to Statistics Sweden (31 December 2021)

Birth Cohort	Total amount of children in the BHVQ N	Percentages of children in Sweden in BHVQ
2011	73	0%
2012	530	0%
2013	1 335	1%
2014	3 623	3%
2015	4 984	4%
2016	6 785	5%
2017	14 713	12%
2018	15 471	13%
2019	14 906	13%
2020	16 701	15%
2021	17 810	16%
2022	14 206	*No denominator available for this birth cohort*
Total	111 137	

Coverage

The completeness of data is compared with data from Statistics Sweden on children born in each region to calculate the coverage (Table 2). The completeness of data differs between regions and birth cohorts. The average coverage for children born in 2021 in participating regions is 71%. Since the children must attend the CHS to be included in the register (information obligatory), a relatively higher proportion of the youngest children are included in the register (Table 2). Children in the BHVQ include those who have had at least one visit to a specific centre in the region at least once. Families have the right to choose to have their child's health care provided in a different region than the one in which they live. Consequently, a total regional coverage does not indicate that all children living in the region have attended a visit in that specific region.

Table 2. Coverage per Region in Sweden. The number of children in each region based on data from Statistics Sweden (31 December 2021) compared with the number of children in the BHVQ per birth cohort. In Stockholm, only one healthcare provider deliver data. In 2023 remaining providers expects to start delivering data.

**Region in**	**Start**	**Birth**	**Children living in**	**Children in the**

Confidential: For Review Only

Sweden	Year and month for delivering data to BHVQ	cohort	the region N	BHVQ N (%)
**Dalarna**	Sep 2017	2021	2 816	2 840 (100)
		2020	2 965	3 026 (100)
		2019	3 059	2 963 (97)
		2018	3 195	2 946 (92)
		2017	3 279	2 824 (86)
**Gotland**	Sep 2022	2021	532	562 (100)
		2020	496	500 (100)
		2019	566	548 (97)
		2018	585	563 (96)
		2017	637	620 (97)
**Gävleborg**	Dec 2021	2021	2 770	1 890 (68)
		2020	2 815	760 (27)
		2019	2 986	439 (15)
		2018	3 276	531 (16)
		2017	3 099	603 (19)
**Jönköping**	Mar 2019	2021	4 054	2 514 (62)
		2020	4 288	3 757 (88)
		2019	4 274	4 020 (94)
		2018	4 416	3 843 (87)
		2017	4 341	3 147 (72)
**Kronoberg**	Oct 2022	2021	2 275	1 949 (86)
		2020	2 402	1 878 (78)
		2019	2 412	1 821 (75)
		2018	2 370	1 844 (78)
		2017	2 398	1856 (77)
**Stockholm**	Nov 2022	2021	28 880	2 905 (10)
		2020	28 288	1 805 (6)
		2019	28 352	2 164 (8)
		2018	28 522	2 470 (9)
		2017	28 968	2 466 (9)
**Södermanland**	Oct 2020	2021	3 113	3 035 (97)
		2020	3 258	3 143 (96)
		2019	3 344	2 719 (81)
		2018	3 593	2 777 (77)
		2017	3 739	2 870 (77)
**Västernorrland**	Oct 2022	2021	2 393	2 178 (91)
		2020	2 325	1 892 (81)
		2019	2 531	293 (12)
		2018	2 658	545 (21)
		2017	2 585	383 (15)

Confidential: For Review Only

Registry data

The registry shows data from child home visits and health outcomes. Table 3 shows some of the most requested data available in the register. Both data on attended home visits, screenings and health outcomes of these can be exported from the register. Data are reported aggregated, but individual data is available for research. In appendix 2 number of children with visits per year and specific age is reported.

Discussion

BHVQ, as part of the local data-driven quality improvement in the CHS

BHVQ allows the CHS to follow and monitor the CHS programme at every clinic. It also enables an automatic collection of data and a data-driven workflow for the CHS. Through the BHVQ, regions can monitor their data continuously, avoid annual reports with outdated data, and compare data nationally. Before BHVQ, annual reports were aggregated regionally with less relevant data for each clinic and could be perceived as criticism and therefore tended to be less of a motivation.[27-29] It also means that there is a risk of receiving feedback far too late on negative trends due to unconscious organisational changes. Using the BHVQ, deviations in the service can be detected early, and relevant interventions can be implemented. The BHVQ provides data to enable data-driven continuous quality improvement within the CHS. With the ability to use the PIN for individuals included in the BHVQ, it is possible to link data to other data sources; moreover, it enables the evaluation of preventive services and their long-term effects. BHVQ can be a tool and a reference for following universal and targeted interventions in the CHS programme.

Several methods used in the CHS programme have limited evidence and are criticized.[30-32] Using BHVQ methods can be evaluated and gain validity or be discarded from the national CHS program. BHVQ can be used to develop and evaluate future methods.

Ethical considerations

Data on health care utilisation and health are sensitive and must be secured. In the BHVQ, the PIN is used as a key; however, when data are exported for research, it must be de-identified or pseudo-anonymised. In register research, data shall be handled with discretion. If the data set contains a large amount of data, the possibility to recognise an individual is minimised. All reporting should be aggregated to avoid the possibility of identifying an individual.

Strengths and Limitations

This study is based on data available in the register. Compared nationally, the coverage in the register is low, but from a regional perspective, most younger cohorts have almost complete coverage. Several regions have ongoing projects to deliver data to the BHVQ, and the coverage for every new birth cohort is increasing. Continuous improvements and data validation create opportunities for the register to increase coverage and widen its usage. The difference in coverage between regions that send data to the BHVQ depends on several factors. All guardians must be informed about the BHVQ before data are transferred to the register. During the child's first year of life, the visits to CHS are more frequent, followed by annual visits. The time delay can be a year or more if the nurse fails to inform the parents about data export to the register. Also, the regional context affects coverage; for example, when electronic health records were implemented.

Confidential: For Review Only

One limitation of this study is that data regarding the quality of child health services and children's health is not analysed. Further studies are needed to evaluate the CHS and give further recommendations regarding changes in the national child health services programme.

Limitations in the documentation and inconsistent documentation are major challenges for the register, together with several active EHR systems in Sweden. Variations in documentation between regions affect the quality of data. For example, the variable regarding exposure to second-hand smoke differs between regions, where some regions report dichotomous variables, and others only document the presence of exposure. The development of the BHVQ must therefore be flexible to include as much data as possible and secure a high-quality level.

Conclusions

This brief description of the data available in BHVQ shows that the BHVQ is a valuable resource for evaluating CHS at a national level. The completeness of data varies between regions and variables and is increasing as more regions deliver data.

As a result of continuous improvement of the services, the possibility to follow the development of children's health in Sweden is possible through BHVQ. When fully expanded, the register will be a natural and essential part of developing preventive services, improving health care for children below six years of age, and a tool for developing evidence-based child health interventions.

Accessing the data online allows decision-makers and healthcare workers to follow and analyse the CHS programme’s outcomes easily. The ambition of the register is that the online platform facilitates resource allocation and improvements to the population’s needs as a data-driven method.

The authors strongly recommend expanding and improving the BHVQ to promote children’s health and develop evidence-based child health care.

Author contribution

MW and AF conceived the study and its design and performed the analyses. MW and AF prepared the draft and AC, AH, AL, FKV, HE, JH, KB, LE, LG MG, SA and TW provided critical revisions of the intellectual content. All authors read and approved the final manuscript.

Funding

The authors have not declared a specific grant for this research from any funding agency in the public, commercial or not-for-profit sectors.

Declaration of interest statement

The authors have no conflicts of interest to declare.

Patient and public involvement

Patients and/or the public were not involved in the design, or conduct, or reporting, or dissemination plans of this research.

The authors would like to thank Meena Strömqvist at Strömqvist Legal & Language Services AB for a good and quick language check.

Confidential: For Review Only

References

1. Wallby T and Hjern A. Child health care uptake among low-income and immigrant families in a Swedish county. *Acta Paediatrica* 2011; 100: 1495-1503. DOI: 10.1111/j.1651-2227.2011.02344.x.
2. Reuter A. Barnhälsovårdens nationella program, https://www.rikshandboken-bhv.se/metoder--riktlinjer/barnhalsovardens-nationella-program/ (2018, accessed 2022-08-09).
3. Nygren US, Sandberg H, Tindberg Y, et al. Perceived needs for team-based visits in Swedish child healthcare services exceed its existence-A mixed-methods study targeting healthcare professionals. *Acta Paediatr* 2022; 111: 653-666. 2021/11/24. DOI: 10.1111/apa.16193.
4. Socialstyrelsen. Vägledning för barnhälsovården, https://www.socialstyrelsen.se/globalassets/sharepoint-dokument/artikelkatalog/vagledning/2014-4-5.pdf (2014).
5. Assembly UNG. Convention on the Rights of the Child. In: Nations U, (ed.). United Nations, Treaty Series1989.
6. Västra Götalandsregionen CB. Årsrapport Barnhälsovård i Västra Götaland. In: Barnhälsovård C, (ed.). Göteborg2018, p. 52.
7. Socialstyrelsen. Vägledning för barnhälsovården. 2014, p. 172.
8. Sveriges Kommuner och Regioner. National system for knowledge-driven management within Swedish healthcare, https://kunskapsstyrningvard.se/kunskapsstyrningvard/omkunskapsstyrning/nationalsystemforknowledgedrivenmanagementwithinswedishhealthcare.56857.html (accessed January 3 2023).
9. Rosén ME, A. Dataregister i vården en kollektiv nyttighet. *Läkartidningen* 1999; 96: 6.
10. Laugesen K, Ludvigsson JF, Schmidt M, et al. Nordic Health Registry-Based Research: A Review of Health Care Systems and Key Registries. *Clin Epidemiol* 2021; 13: 533-554. 2021/07/30. DOI: 10.2147/CLEP.S314959.
11. Ludvigsson JF, Otterblad-Olausson P, Pettersson BU, et al. The Swedish personal identity number: possibilities and pitfalls in healthcare and medical research. *Eur J Epidemiol* 2009; 24: 659-667. 2009/06/09. DOI: 10.1007/s10654-009-9350-y.
12. Regulation (EU) 2016/679 of the European Parliament and of the Council of 27 April 2016 on the protection of natural persons with regard to the processing of personal data and on the free movement of such data, and repealing Directive 95/46/EC (General Data Protection Regulation). *Official journal of the European Union*; 59.
13. Patientdatalag (2008:355). In: Socialdepartementet, (ed.). Svensk Författningssamling (SFS).
14. Lindfors A and Almqvist-Tangen G. Hembesök - Rikshandboken för Barnhälsovård, https://www.rikshandboken-bhv.se/metoder--riktlinjer/hembesok/ (2021, accessed 2022-06-01).
15. Barboza M, Kulane A, Burstrom B, et al. A better start for health equity? Qualitative content analysis of implementation of extended postnatal home visiting in a disadvantaged area in Sweden. *Int J Equity Health* 2018; 17: 42. 2018/04/11. DOI: 10.1186/s12939-018-0756-6.
16. Barboza M, Marttila A, Burstrom B, et al. Towards health equity: core components of an extended home visiting intervention in disadvantaged areas of Sweden. *BMC Public Health* 2022; 22: 1091. 2022/06/02. DOI: 10.1186/s12889-022-13492-3.
17. Burström B, Marttila A, Kulane A, et al. Practising proportionate universalism - a study protocol of an extended postnatal home visiting programme in a disadvantaged area in Stockholm, Sweden. *BMC Health Serv Res* 2017; 17: 91. 2017/01/29. DOI: 10.1186/s12913-017-2038-1.
18. Cox JL, Holden JM and Sagovsky R. Detection of postnatal depression. Development of the 10-item Edinburgh Postnatal Depression Scale. *Br J Psychiatry* 1987; 150: 782-786. 1987/06/01. DOI: 10.1192/bjp.150.6.782.

Confidential: For Review Only

19. Derwig M. Instruktioner för mätning och vägning på BVC, https://www.rikshandboken-bhv.se/tillvaxt--utveckling/tillvaxt/instruktioner-for-matning-och-vagning-pa-bvc/ (accessed 2022-09-05).
20. Cole TJ and Lobstein T. Extended international (IOTF) body mass index cut-offs for thinness, overweight and obesity. *Pediatr Obes* 2012; 7: 284-294. DOI: 10.1111/j.2047-6310.2012.00064.x.
21. Mattsson CM, Marild S and Pehrsson NG. Evaluation of a language-screening programme for 2.5-year-olds at Child Health Centres in Sweden. *Acta Paediatrica* 2001; 90: 339-344.
22. Westerlund M and Sundelin C. Can severe language disability be identified in three-year-olds? Evaluation of a routine screening procedure. *Acta Paediatrica* 2000; 89: 94-100. 2000/02/17.
23. Cook DG and Strachan DP. Health effects of passive smoking-10: Summary of effects of parental smoking on the respiratory health of children and implications for research. *Thorax* 1999; 54: 357-366. 1999/03/27. DOI: 10.1136/thx.54.4.357.
24. Makadia LD, Roper PJ, Andrews JO, et al. Tobacco Use and Smoke Exposure in Children: New Trends, Harm, and Strategies to Improve Health Outcomes. *Curr Allergy Asthma Rep* 2017; 17: 55. 2017/07/26. DOI: 10.1007/s11882-017-0723-0.
25. Wallby T and Hjern A. Parental region of birth, socio-economic status and infants' exposure to second-hand smoke. *Acta Paediatr* 2008; 97: 1542-1545. 2008/08/16. DOI: 10.1111/j.1651-2227.2008.00964.x.
26. Meek JY, Noble L and Section on B. Policy Statement: Breastfeeding and the Use of Human Milk. *Pediatrics* 2022; 150 2022/08/04. DOI: 10.1542/peds.2022-057988.
27. Arvidsson E, Dahlin S and Anell A. Conditions and barriers for quality improvement work: a qualitative study of how professionals and health centre managers experience audit and feedback practices in Swedish primary care. *BMC Fam Pract* 2021; 22: 113. 2021/06/16. DOI: 10.1186/s12875-021-01462-4.
28. Regeringskansliet. SOU 2021:34, Börja med barnen! - En sammanhållen god och nära vård för barn och unga. Delbetänkande av Utredningen om en sammanhållen god och nära vård för barn och unga. In: Socialdepartementet, (ed.). Stockholm: Statens offentliga utredningar, 2021, p. 684.
29. Regeringskansliet. SOU 2021:78, Börja med barnen! - Följa upp hälsa och dela information för en god och nära vård. Slutbetänkande av Utredningen om en sammanhållen god och nära vård för barn och unga. In: Socialdepartementet, (ed.). Stockholm: Statens offentliga utredningar, 2021, p. 272.
30. Siu AL, on behalf of the USPSTF and Force USPST. Screening for Speech and Language Delay and Disorders in Children Aged 5 Years or Younger: US Preventive Services Task Force Recommendation Statement. *Pediatrics* 2015; 136: e474-e481. DOI: 10.1542/peds.2015-1711.
31. Cox J. Thirty years with the Edinburgh Postnatal Depression Scale: voices from the past and recommendations for the future. *The British Journal of Psychiatry* 2019; 214: 127-129. 2019/02/18. DOI: 10.1192/bjp.2018.245.
32. UK National Screening Committee. Child screening programme Obesity, https://view-health-screening-recommendations.service.gov.uk/obesity/ (2018, accessed January 3 2023).

Confidential: For Review Only

Table 3. Results of activities and measurements in the CHS that are exported to the BHVQ. In percentages

Birth Cohort	Received home visits	Received EPDS	Children who got breast milk at 4 months of age	Exposure to second hand smoke at 8 months of age; mother smokes	Exposure to second hand smoke at 8 months of age; father smokes	Positive on language screening at 2.5–3 years of age	Negative on language screening at 2.5–3 years of age	No completed language screening at 2.5–3 years of age	Underweight at 2.5–3 years of age	Overweight at 2.5–3 years of age	Obesity at 2.5–3 years of age	Underweight at 4 years of age	Overweight at 4 years of age	Obesity at 4 years of age
**2011**									0	0	0	3	13	2
**2012**						20	80	16	1	14	6	3	13	5
**2013**						17	83	3	1	21	5	1	13	4
**2014**				3,9	7,1	15	85	6	2	18	4	1	12	4
**2015**	55	59	72	5,2	8,5	16	84	32	2	17	3	2	10	3
**2016**	70	65	70	6,2	8,4	14	88	11	2	16	3	2	11	3
**2017**	75	70	71	3,4	6,5	12	90	26	2	18	4	2	12	4
**2018**	72	51	72	3,9	7,2	12	88	20	2	17	4	2	10	3
**2019**	78	64	71	3,6	7,5	13	87	11	2	14	3	0	0	0
**2020**	63	79	72	2,9	6,2	16	85	6	1	12	2	0	0	0
**2021**	74	76	69	2,7	5,4				0	0	0	0	0	0
**2022**	79	73	71	1,6	3,7				0	0	0	0	0	0

Confidential: For Review Only

Variable	Description	Age of measurements
First born child	Is the child the first born child for any caregiver	0-5 years old
Home visits	Received home visits, per child. Two home visits are a part of the national program from child health care	0-5 years old
Visits at the clinics	Visits at the CHC, per child. Presented per profession the child met at the visits (nurse, physician, paediatrician and GP)	0-5 years old
Team visits at the clinics	Children who meet both a physician and a specialist nurse at the CHC	0-5 years old
Parental support in group	Children whose caregivers participated in a parental support group at the CHC. The groups are usually conducted during the child's first year.	0-5 years old
Postnatal Depression Screening	Children where the mother/birth-giving parent participated in screening for postnatal depression using the Edinburgh Postnatal Depression Scale (EPDS)	6-8 weeks
Individual conversation with the non birth giving caregiver	Children where the father/non-birth-giving parent participated in an individual conversation at the CHC regarding mental health and parenthood	3-5 months
Breastfeeding status	Breastfeeding status during the first year with measurements after 1 week, 2, 4, 6, 8 and 12 months	0-12 months
Vaccinations	Number of doses per child and vaccine in the national vaccination programme for children. This includes vaccinations against rotavirus infection, diphtheria, tetanus, pertussis, polio, Haemophilus influenzae type b, pneumococcal disease, measles, mumps, and rubella. For children "at-risk" the vaccination program also includes selective vaccination against tuberculosis, hepatitis B, influenza and invasive pneumococcal disease, for BHVQ tuberculosis and hepatitis B is included	0-5 years old. National measurement standard at 2 years
Second-hand smoking	Exposure to second hand smoke by caregivers who smokes or the occurrence of smoking in the child's home	4 weeks, 8 months and 18 months
Growth	Weight, Length/Height, Head Circumference (during the first 18 months), Gestational age at birth and biological parents length. Body mass index (BMI) is calculated at 2,5-3 years, 4 years and 5 years	0-5 years old
Language and communication screening	Completed, no need for assessment and result of screening	2,5-3 years
Audiology screening	Completed, no need for assessment and result of screening	4 years
Ophthalmic screening	Completed, no need for assessment and result of screening	4 years
Developmental assessment	Completed, no need for assessment and result of screening	4 years

Confidential: For Review Only

Referrals	Referrals send from CHC to audiologist, paediatrician, physiotherapist, speech and language pathologist, orthoptist and psychologist per child	0-5 years old
Date of discharge from delivery ward and maternity ward	Date of discharge from delivery ward and maternity ward	0-3 months
Accidents	Indicators of pre-school children's health	12 months and 4 years
Antibiotics	Indicators of pre-school children's health	12 months and 4 years
Asthma	Indicators of pre-school children's health	12 months and 4 years
Eczema	Indicators of pre-school children's health	12 months and 4 years

Confidential: For Review Only

Appendix 2. The total amount of children in the register with visits at a specific age

Birth Cohort	Total amount of children in the BHVQ	Children with at least one visit at age 0-3 weeks (0–18 days)	Children with at least one visit at age 0-6 weeks (0–42 days)	Children with at least one visit at age 0 weeks - 2 months and 3 weeks (0–82 days)	Children with at least one visit at 4 months of age 105–143 days	Children with at least one visit at 6 months of age (167–213 days)	Children with at least one visit at 8 months of age (227–274 days)	Children with at least one visit at 1,5 years of age (517–638 days)	Children with at least one visit at 2,5-3 years of age (853–1186 days)	Children with at least one visit at 4 years of age (1401–1551 days)	Children with at least one visit at 5 years of age (1766–2100 days)
2011	73									62	73
2012	530		1	1	1	1	1	1	161	409	545
2013	1 335	1	1	1	1	2	1	167	982	1 096	1 304
2014	3 623	78	99	105	116	259	411	1 132	1 382	2 288	3 575
2015	4 984	827	965	1 089	1 171	1 151	1 240	1 395	2 871	4 438	4 786
2016	6 785	1 945	2 094	2 141	1 909	2 058	2 062	2 446	5 345	5 618	6 116
2017	14 713	4 619	5 093	5 274	4 833	5 277	5 554	8 103	10 886	11 585	11 819
2018	15 471	6 541	7 199	7 568	7 503	8 467	8 738	10 373	13 124	11 797	26
2019	14 906	9 543	10 104	10 428	9 737	10 225	10 297	11 715	12 449	28	
2020	16 701	12 973	13 659	13 978	12 942	13 363	13 151	13 685	4 910		
2021	17 810	14 013	15 023	15 641	13 896	14 567	14 493	6 671			
2022	14 206	12 982	13 907	14 145	8 464	6 736	4 154				
Total	111 137	63 522	68 145	70 371	60 573	62 106	60 102	55 688	52 110	37 321	28 244

Confidential: For Review Only
